# Nose-to-Brain Delivery of Antiviral Drugs: A Way to Overcome Their Active Efflux?

**DOI:** 10.3390/pharmaceutics10020039

**Published:** 2018-03-26

**Authors:** Alessandro Dalpiaz, Barbara Pavan

**Affiliations:** 1 Department of Chemical and Pharmaceutical Sciences, University of Ferrara, 44121 Ferrara, Italy; 2 Department of Biomedical and Specialist Surgical Sciences, University of Ferrara, 44121 Ferrara, Italy; pvnbbr@unife.it

**Keywords:** active efflux transporter, antiretroviral drug, HIV sanctuaries, thermoreversible gel, nano-emulsion, polymeric microparticles, polymeric nanoparticles, nasal formulation, solid lipid microparticles, virus

## Abstract

Although several viruses can easily infect the central nervous system (CNS), antiviral drugs often show dramatic difficulties in penetrating the brain from the bloodstream since they are substrates of active efflux transporters (AETs). These transporters, located in the physiological barriers between blood and the CNS and in macrophage membranes, are able to recognize their substrates and actively efflux them into the bloodstream. The active transporters currently known to efflux antiviral drugs are P-glycoprotein (ABCB1 or P-gp or MDR1), multidrug resistance-associated proteins (ABCC1 or MRP1, ABCC4 or MRP4, ABCC5 or MRP5), and breast cancer resistance protein (ABCG2 or BCRP). Inhibitors of AETs may be considered, but their co-administration causes serious unwanted effects. Nasal administration of antiviral drugs is therefore proposed in order to overcome the aforementioned problems, but innovative devices, formulations (thermoreversible gels, polymeric micro- and nano-particles, solid lipid microparticles, nanoemulsions), absorption enhancers (chitosan, papaverine), and mucoadhesive agents (chitosan, polyvinilpyrrolidone) are required in order to selectively target the antiviral drugs and, possibly, the AET inhibitors in the CNS. Moreover, several prodrugs of antiretroviral agents can inhibit or elude the AET systems, appearing as interesting substrates for innovative nasal formulations able to target anti-Human Immunodeficiency Virus (HIV) agents into macrophages of the CNS, which are one of the most important HIV Sanctuaries of the body.

## 1. Viruses Can Have Important Neurotropic Effects

It is currently well-known that several viruses can have important neurotropic effects in infected bodies: as an example, Canine Distemper Virus (CDV) and Measles Virus (MV) are known to cause demyelinating disease of the central nervous system (CNS) in dogs and humans, respectively; and Eastern Equine Encephalitis Virus (EEEV) is able to induce death or long-lasting and severe neurological sequelae in humans [1]. Even if Vesicular Stomatitis Virus (VSV) is considered promising as a vaccine vector, its greatest limitation is potential neurotropic activity that can be lethal within the brain [2,3,4]; moreover, Herpes Simplex Virus type 1 (HSV-1) can cause potentially fatal encephalitis in developed countries [5,6]. Finally, mice intra-nasally infected by Venezuelan Equine Encephalitis Virus (VEEV) show a CNS phase that results in encephalitis and death [7,8,9].

Human Immunodeficiency Virus type-1 (HIV-1) belongs to the lentivirus family. It displays a long latency period and a slow progressive disease culminating in severe immune deficiencies together called acquired immune deficiency syndrome (AIDS) [10]. The virus firstly infects CD4^+^ T lymphocytes, causing a severe drop in their immune effector functions. Moreover, HIV-1 shows long-term persistence in monocytes that can easily enter the CNS across the blood–brain barrier (BBB), where they differentiate in macrophages that are known to harbor and replicate the virus [11,12]. The presence of HIV in the brain can lead to dementia in the more severe cases [13,14]. The CNS therefore constitutes one of the sanctuaries for HIV, from which the periphery can be re-infected and where drug resistance is induced [11,15]. Indeed, despite the fact that antiretroviral therapies are widely used in the treatment of AIDS and that their administration dramatically reduces viral loads in HIV patients, the eradication of the virus from the HIV sanctuaries cannot be obtained since the drugs are unable to reach them with therapeutic concentrations [16]. Currently, the guidelines of the Department of Health and Human Services for the use of antiretroviral agents in adults and adolescents living with HIV indicate several combination-based regimens using antiretroviral drugs belonging to mechanistic classes that include nucleoside/nucleotide reverse transcriptase inhibitors (NRTIs), non-nucleoside reverse transcriptase inhibitors (NNRTIs), protease inhibitors (PIs), a CCR5 antagonist, such as maraviroc, or integrase inhibitors (INs), such as dolutegravir [17,18,19,20]. It is important to remark that long-term exposure to high doses of anti-HIV drugs, in order to try to enhance their uptake in HIV sanctuaries, can cause severe side effects, such as lipodystrophy, diabetes, and cardiovascular disease [21].

## 2. Active Efflux Transporters Do Not Allow the Antiviral Drugs to Reach the Sanctuaries of Viruses

The lack of penetration of antiretroviral drugs in the HIV sanctuaries is mainly due to the expression of active efflux transporters (AET) on the membranes of lymphocytes [22,23,24], macrophages [25], and the cells that constitute the blood−brain (BBB) and blood−cerebrospinal fluid (BCSFB) barriers [26,27,28]. The AET systems can be members of two transporter gene superfamilies [28,29,30,31]:

- The ATP-binding cassette (ABC) gene family of active transporters requiring ATP hydrolysis for their efflux activity; and
- The solute carrier (SLC) gene family of energy-independent or secondary active efflux transporters.

As far as antiretroviral drugs are concerned, the most studied AET systems belong to the ABC gene family; in particular, these are P-glycoprotein (P-gp-ABCB or MDR gene family), multidrug resistance–associated proteins (MPRs-ABCC gene family), and breast-cancer-resistance protein (BCRP-ABCG gene family) [24,32,33,34,35,36,37,38].

The substrates of P-gp are amphipatic cations and organic compounds whose molecular weights range from 200 to almost 1900 Da [24], so P-gp shows a broad substrate spectrum. MRPs transport hydrophilic anion compounds or large molecules (MRP-1) [39] or small polar compounds (for example, nucleosides), cyclic nucleotides, and nucleoside analogues (MRP-4, MRP-5) [40]. The substrate specificity of BCRP appears similar to that of P-gp [27].

Table 1 reports the AET subtypes currently known to interact with antiviral drugs. It is evidenced that the clinically approved PIs, saquinavir, ritonavir, and lopinavir, are substrates of both P-gp and MRP-1 [24,35,37,41,42]. The antiviral drugs amprenavir, nelfinavir, indinavir (PIs), and abacavir (NRTI) are known as P-gp substrates [43], whereas zidovudine and didanosine appear to be transported by MRP-4 and MRP-5 [32,33,38]. Finally, zidovudine, lamivudine, abacavir, zalcitabine, stavudine, and efavirenz appear as BCRP substrates [44], whereas ritonavir, saquinavir, and nelfinavir are known as inhibitors of this transporter [34]. Maraviroc, a CCR5 inhibitor, is suggested to be a P-gp substrate [45,46,47], whereas the IN dolutegravir appears as a P-gp and BRCP substrate [20]. Cyclosporine-A, verapamil, and mefloquine are P-gp inhibitors; some of them have been used to define an MDR-1-specific efflux of antiviral drugs [41,48,49]. Finally, paclitaxel, probenecid, and the leukotrienes (LT) receptor antagonist, e.g., MK-571, act as inhibitors of MRPs [38,39,40,50].

## 3. Antiviral Drugs Can Enhance the Expression of Active Efflux Transporters

It is known that exposure to xenobiotic drug substrates can enhance the expression of active efflux transporters on brain microvascular endothelial cells. As an example, it has recently been reported that the concomitant exposition of primary human brain microvascular endothelial cells (HBMVEC) to HIV-1 and saquinavir induces an increased MDR-1-mediated drug efflux [51]. Moreover, it has been demonstrated that the PIs ritonavir and atazinavir are able to induce P-gp expression in brain microvessel endothelial cells belonging to the BBB [52,53,54]. These phenomena thus involve a further restriction to the entry of antiretroviral drugs to the central nervous system.

The induction of P-gp in peripheral organs and brain microvessel endothelial cells appears to be mediated through the activation of the nuclear pregnane X (PXR) and constitutive androstane (CAR) receptors [55,56,57,58,59,60]. In particular, the human receptors hPXR and hCAR appear actively involved in the regulation of P-gp expression in the human brain microvessel endothelial cell culture system [61]. A few PIs, such as ritonavir, amprenavir, and lopinavir, are known to be hPXR ligands or activators [62,63]. Recently, in vitro studies performed on human brain microvessels endothelial cells hCMEC/D3 demonstrated that amprenavir, atazanavir, efavirenz, ritonavir, and lopinavir activate hPXR, whereas abacavir, efavirenz, and nepvirapine activate hCAR. Moreover, these drugs appear to be able to increase P-gp expression in hCMEC/D3 cells [64]. In other words, a great number of PIs are hPXR ligands: abacavir and nevirapine are hCAR ligands, and efavirenz is a ligand of both PXR and hCAR. These data suggest that the nuclear receptor activity of these ligands can further restrict their ability to enter the brain, being able to increase P-gp expression at the BBB level. Taking these aspects into account, it has been suggested that the targeted suppression of P-gp expression in the HIV-1-infected reservoirs of the body may constitute a new strategy able to inhibit antiretroviral drug efflux from the brain [51].

## 4. AET Inhibitors: Promising in Vitro Results Not Confirmed by Clinical Trials

The first AET inhibitors were discovered about 30 years ago when the enhancement of the cytotoxicity of some anticancer drugs induced by verapamil (a vasodilator) and cyclosporin A (an immunosuppressant) was revealed. These drugs were indeed able to reverse the overexpression effects of P-gp. However, an important obstacle related to this type of inhibitor was the very high concentration required to inhibit P-gp, since they were not specifically designed to be inhibitors of efflux transporters. The high concentrations of inhibitors induced severe unwanted effects when their distribution was ubiquitous in the body. As a consequence, this “first-generation” of inhibitors could not be used in clinical trials [65,66]. Analogs of first-generation inhibitors were obtained as a “second generation” of inhibitors (such as valspodar), which were characterized by higher inhibitory activity and the absence of therapeutic targets other than the targeted transporters. However, also in this case, patients suffered severe unwanted side effects following their administration, which were likely due to their pharmacokinetic interactions with the drugs [28,65,66,67]. Finally, a “third generation” of AET inhibitors (such astariquidar, zosuquidar, and laniquidar), which was characterized by very high potency and the absence of drug metabolic interactions, was not confirmed as being deprived of severe unwanted effects when administered to patients, probably as a result of their ubiquitous activity on the cells of the body [28,65,66,67].

## 5. Prodrugs of Antiviral Drugs: New Proposals against the AET Activity

Taking into account the difficulties related to the clinical use of ABC transporter inhibitors, the design of prodrugs able to elude the transporters would appear to be a promising solution to this problem. Moreover, new formulations able to target the drugs in the CNS bypassing the BBB are emerging. The loading of ABC transporter inhibitors in this last type of formulation could open interesting perspectives for the selective target of their action within the central nervous system [28].

Knowledge of the structure–activity relationships (SAR) of ABC efflux pumps should be necessary for a prodrug approach, but, in general, the AET systems are characterized by multiple binding sites and do not interact with their substrates in a lock-and-key manner, so their activity cannot be readily evaluated by classic Michaelis–Menten kinetics [27,68]. A first SAR approach in order to obtain antiviral prodrugs able to inhibit AET efflux activity was proposed by Namanja and co-workers [26] on the basis of the solved crystal structure of mouse P-gp [69]. Taking into account that P-gp shows a large and fluid binding cavity, the HIV reverse transcriptase inhibitor abacavir was converted into a dimeric prodrug able to act as a potent P-gp inhibitor and revert to the monomeric form upon entry into cells overexpressing the efflux transporter. The tether length between the abacavir molecules in the prodrug dimers was able to modulate their inhibitory potency in two different cell lines, in particular, a human brain capillary endothelial cell line expressing endogenous levels of P-gp and a P-gp overexpressing CD4^+^ T-lymphocyte cell line [70]. The approach of dimeric prodrugs as AET inhibitors could be interesting, as selective targeting of the prodrugs may be obtained in the central nervous system where it seems reasonable to hypothesize they can easily reach therapeutic concentrations in the cells acting as HIV reservoirs.

Recently, we proposed a new prodrug strategy of antiviral drugs demonstrating that the ester conjugation of zidovudine (AZT) with ursodeoxycholic acid, a bile acid which can permeate the CNS, results in a prodrug (UDCA–AZT) able to elude the AET transporters whose AZT is a substrate in cell monolayers showing epithelial barrier features [71]. In particular, this type of prodrug was not effluxed from cell monolayers able to efflux AZT, but, at the same time, the activity of the transporters was not inhibited by the prodrug itself [71]. These data suggest that the conjugation of antiviral drugs with bile acids may constitute a new strategy in order to elude, without inhibiting, the AET systems that normally preclude the entry of the antiretroviral drugs in HIV sanctuaries. In this regard, we have very recently confirmed that UDCA–AZT is able to permeate and remain in murine macrophages with an efficiency which is twenty times higher than that of AZT [25]. Moreover, the prodrug, obtained by conjugation of AZT with UDCA, appeared to be suitable for loading in both polymeric micro- or nano-spheres and solid lipid microparticles [25,72,73] used as innovative formulations to target the prodrug in the central nervous system following their nasal administration. The aspects regarding brain targeting following nasal administration of antiviral drugs or their prodrugs will be discussed in the following sections.

## 6. Micro- and Nano-Particulate Systems: Can These Innovative Formulations Target the Antiviral Drugs in the Central Nervous System?

Bearing in mind that monocytes and macrophages can act as Trojan Horses for all members of the lentivus family (thus inducing the entry of HIV into its sanctuaries), using the migratory ability of these cells has been proposed in order to carry the antiretroviral drugs to the central nervous system [74]. In particular, the idea of formulating nanoparticles loaded with an antiretroviral drug has been developed in order to obtain their efficient takeup by macrophages, that following intravenous administration, should migrate to the HIV-infected tissues and release the antiretroviral drug. In this regard, it has been reported that indinavir (IDV)-loaded nanoparticles, obtained by coating an IDV suspension, prepared by high-pressure homogenization, with Lipoid E80, were efficiently uptaken by virus infected macrophages. The loaded nanoparticles were able to suppress virus replication for a long period of time in the macrophages [75]. The intravenous administration of macrophages containing the nanoparticles were shown to migrate to the lung, spleen, liver, and lymph nodes, where they induced strong antiretroviral activities [76]. As far as the CNS was concerned, other strategies to target the antiviral drugs appeared to be more suitable.

## 7. Nasal Administration: A Promising Strategy for Antiviral Drug Uptake in the Brain

Receptor-mediated transportation, transporter-mediated transport, adsorptive-mediated transportation, or temporarily increased BBB permeability represent several important strategies currently studied in order to obtain the uptake of drugs in the CNS. Receptor-mediated transportation is based on the binding of specific receptors of BBB by ligands able to mediate their internalization into cells. This strategy is often related to high costs of formulation [77].

Transporter-mediated transport is based on the conjugation of drugs with nutritive material molecules that can be recognized and taken up in the CNS by specific transporters overexpressed in the BBB. Unfortunately, several conjugates may not be taken up even if they are recognized by the transporters [78]. Adsorptive-mediated transportation is based on the use of cationic proteins and peptides which can interact with negatively charged BBB. This strategy is, however, affected by poor selectivity toward the CNS [77]. A temporary opening of the BBB can be obtained by chemical compounds able to enhance BBB permeability or by receptor-involved changing of tight junctions; in this case, well-optimized protocols are needed [77].

In order to obtain the uptake of antiviral drugs in the central nervous system, the nasal approach would appear to be a promising strategy, as it is potentially able to deliver drugs directly into the CNS from the nasal cavity [77]. Intranasal delivery was indeed discovered about 30 years ago as a new method for delivery of drugs to the CNS [74]. Currently, it is well-known that after nasal application, a drug-escaping mucociliary clearance and enzymatic degradation can permeate not only into the bloodstream, but also into the cerebrospinal fluid (CSF) or the brain tissue across the olfactory region or via the trigeminal pathway of the nasal cavity [79,80]. There are three known pathways allowing the drug access to the brain or CSF from the nasal cavity: (i) the olfactory pathway (direct paracellular or transcellular transport via the olfactory neurones or olfactory epithelial cells); (ii) the trigeminal pathway (transport via trigeminal nerves); and (iii) the systemic pathway (the drug is absorbed into the bloodstream, then it has the ability to cross the BBB) [81]. The last of these pathways appears to have the poorest chances of allowing antiviral drugs access to the central nervous system, since most of them are substrates of the AET systems expressed in the BBB. In contrast, the olfactory and trigeminal pathways seem to offer better chances of the antiviral drugs being targeted in the brain. It is indeed known that drug delivery from the nose to the CNS along these pathways can occur within a few minutes by an extracellular route without binding to any receptor or undergoing axonal transport [82]. Some drugs can be axonally transported into the brain after endocytosis, but this process generally requires a few days [74]. The olfactory neural pathway allows for distribution of the drugs in the olfactory bulb, anterior olfactory nucleus, frontal cortex, and hippocampus (rostral brain structures); the trigeminal pathway allows for drug distribution in the upper cervical spinal cord, midbrain, pons, and hypothalamus (caudal brain structures) [79].

The efficacy of nasal administration against neurotropic viral effects was tested on mice infected by herpes simplex virus type 1 (HSV-1). In particular, mice models of HSV-1 encephalitis received an intranasal pretreatment with the immunostimulant polyinosinic:polycytidylic acid (poly I:C) using a saline vehicle, and they showed a significantly higher mean life expectancy and an increased rate of survival than mice with an intraperitoneal pretreatment of poly I:C [83].

The nasal administration of a water solution of ribavirin, a nucleoside analogue able to inhibit the canine distemper virus (CDV) and also indicated for treatment of hepatitis C [84], allowed investigators to obtain drug concentrations in the olfactory bulb of rats similar to those obtained after intravenous administration, whereas the nasal administration of the raw solid drug, using a specific Dry-Powder Insufflator designed to produce a puff of fine powder, led to significantly higher levels [85]. This result allows us to hypothesize that formulations able to increase the contact between the drug and olfactory nasal mucosa may induce an enhancement of drug bioavailability in the brain. Very recently, a powder formulation of agglomerates constituted by micronized ribavirin and α-cyclodextrin spray-dried microparticles was nasally administered to rats. Ribavirin accumulation in the brain obtained by this formulation was higher than that obtained by nasal administration of ribavirin-micronized powder in the absence of α-cyclodextrin microparticles able to induce penetration-enhancing properties [86]. Appropriate strategies therefore appear necessary for both formulations and devices in order to optimize drug uptake in the CNS after nasal administration.

## 8. What Strategies Are Currently Related to Nasal Administration of Antiviral Drugs?

It is important to underline that effective brain uptake requires special devices and nasal formulations able to induce drug deposition in the olfactory region of the nose, prolonged residence time, and high local drug concentration for diffusion. Several strategies appear necessary in order to optimize brain delivery of drugs by intranasal administration, such as the addition of penetration enhancers or mucoadhesive materials or the preparation of micro- and nano-particulate formulations [87].

### 8.1. An Innovative Device for the Nasal Administration of Antiviral Drugs

Many devices for nasal administration are known, even if not all appear to be suitable for specific deposition of drug amounts in the olfactory region of the nasal cavity. As an example, spray pumps, drops and syringes with tubes, or a vortical flow atomizer are not recommended for nose-to-brain delivery of drugs, whereas appropriate pressurized metered dose inhalers are designed for this purpose. Recently, a bidirectional breath-powered nasal delivery platform was proposed in order to optimize the deposition of drugs in the nasal cavity and nose-to-brain delivery while overcoming unwanted pulmonary distribution [88,89,90]. Concerning innovative devices for the nasal administration of antiviral drugs, a pressurized olfactory delivery (POD) aerosol was developed in order to induce the deposition of a greater amount of drugs into the olfactory region of the nasal cavity in rats [88]. Two model drugs, mannitol and relfinavir, were chosen in order to compare their brain and blood levels after nasal administration of nose drops in rats, which deposited primarily on the respiratory region, or after deposition primarily on their olfactory region with a POD. The cortex-to-blood ratio increased about 4- and 16-fold for mannitol and relfinavir, respectively, with POD administrations compared to those with nose drops. This result confirms that an efficient and selective deposition of drugs on the olfactory region of the nasal cavity induces efficacious direct nose-to-brain transport [88].

### 8.2. Design of Innovative Nasal Formulations for Antiviral Drugs

Currently, the clinical nasal formulations involving antiviral effects appear to be focused on vaccines [91] or on treatments against cold viruses acting outside of the central nervous system [92]. Innovative formulations able to target antiviral drugs in the CNS are therefore needed. Regarding studies on innovative nasal formulations, the development of antiretroviral efavirenz (EFV)-loaded nanoparticles based on poly(ε-caprolactone) (PCL), Eudragit**^®^** RS 100 (RS, a copolymer of ethylacrylate, methylmethacrylate, and methacrylic acid esterified with quaternary ammonium groups), and their blends has been proposed [93]. The nanoparticles were obtained by nanoprecipitation or emulsion/solvent diffusion/evaporation, and nanoprecipitation was shown to result in smaller particles and narrower size distribution patterns, even if all of the systems displayed a remarkably high encapsulation efficiency and drug payload regardless of the polymer composition and the production technique. The poly(methacrylate)’s presence in formulation procedures allowed for a fine tuning of the particle size and the release kinetics; moreover, the incorporation of poly(methacrylate) induced strongly positive Z-potential values of the nanoparticles [93]. This property should induce mucoadesive properties in the nanoparticles with a likely improvement in their residence time in contact with the nasal mucosa. It is indeed well-known that the presence of positive charges induces mucoadhesive properties in polymers, since the mucosal membranes are characterized by the presence of negatively charged species [94].

It is important to remark that an efficient nasal formulation should be able to prolong the residence time within the nasal cavity in order to enhance the bioavailability of the drug. This goal can be achieved with the use of “intelligent” polymers, which may respond to nasal cavity temperature [95] or pH [96], or which can show mucoadhesive properties that enhance the adhesion of formulations onto nasal mucosa [97] and drug bioavailability [98].

Thermoresponsive and mucoadhesive hydrogel formulations based on the thermosensitive polymer Pluronic**^®^** 127 and either carboxymethyl cellulose or chitosan were studied in vitro as intranasal sprays in order to optimize their deposition in the nasal cavity, their gel transition at 34 °C (the nasal cavity temperature), and the release profiles of adamantine chosen as a model drug [99]. Studies performed using an in vitro human nasal airway model evidenced that chitosan can provide the requirements for mucoadhesion in these formulations [99].

Chitosan is a cationic polysaccharide obtained from the deacetylation of chitin, a polymer abundant in cretaceous organisms. Chitosan is currently known for its biocompatibility, biodegradability, and low toxicity, and is therefore used in several pharmaceutical applications [100]. Chitosan appears to be promising for nasal delivery, as it is able to bind to the nasal mucosal membrane inducing an increase in the retention time of formulations which contain it. Indeed, the positively charged amino groups of chitosan effectively interact with the anionic groups of the mucous layers [81]. Moreover, chitosan appears to be a good absorption enhancer, as it is able to transiently open the tight junctions in the epithelial cells [101]. These properties allow chitosan to prolong the residence time of a formulation in the nasal cavity and, at the same time, to enhance drug permeation across the mucosal membranes [81].

Among the biocompatible polymers, poly-*N*-vinyl-2-pyrrolidone (PVP) shows a wide range of pharmaceutical applications as a hydrogel, including in nasal formulations [102]. Blends of PVP and polyethylene glycol (PEG) 600 show adhesive properties [103].

Polymeric mucoadhesive hydrogels based on PVP or chitosan have been studied as nasal delivery formulations for acyclovir. In particular, the muco-adhesivity was studied in the nasal mucosal tissue of sheep by evaluating the force required to detach the formulations from the mucosa. The most mucoadhesive formulations were shown to be chitosan gel and PVP in the presence of PRG 600. Higher release rates were evidenced by PVP compared to chitosan gels [104].

In addition to chitosan, papaverine (a phosphodiesterase inhibitor) has also been considered for its action on tight junctions of nasal mucosa. In particular, after intranasal administration to rats, papaverine appeared to be able to induce a rapid and transient decrease in the tight junction protein-phosphorylated occludin in the olfactory epithelium. This phenomenon was associated with an approximately four-fold increase in the amounts of gemcitabine (chosen as a model drug) reaching the brain, which was explained by transient dephosphorylation of occludin following disassembly of the tight junctions in the cytoplasm of mucosal cells [105].

### 8.3. Nasal Formulations and Brain Targeting of Antiviral Drugs

New nasal formulations of antiviral drugs have been administered in vivo in order to evaluate drug absorption in the central nervous system. Among these, an intranasal nano-emulsion based on the hydrophobic oil Capmul MCM, in the presence of Tween 80 and PEG 400 as surfactant and co-surfactant, respectively, was developed for brain targeting of saquinavir mesylate. This type of formulation was able to induce a higher concentration of the drug in the brain after intranasal administration compared to saquinavir amounts obtained after intravenous administration of plain drug suspension. In particular, the drug targeting efficacy (DTE) and nose-to-brain drug direct transport percentage (DTP) values obtained by nasal administration of the nano-emulsion were 2919 and 97, respectively, indicating the efficacy of this formulation in promoting the nose-to-brain delivery of saquinavir mesylate [106].

#### 8.3.1. Nasal Formulations for Zidovudine Administration

Several nasal formulations have been studied in order to induce the uptake of zidovudine (AZT) in the central nervous system. It is well-known that AZT penetration in the CNS is poor because it is a substrate of AET systems expressed by both the BBB and BCSFB [107,108,109]. The AET systems induce, in vivo, an asymmetric transport of AZT across these barriers, where the rate of AZT efflux from the CNS to blood is greater than its influx rate [110], resulting in a poor CNS/plasma ratio (about 0.1) of zidovudine [111]. It is important to underline that AZT activity in the CNS is of great importance in those CSF subarachnoid spaces that contain macrophages constituting the only site of HIV replication in the brain [112,113]. The presence of AET systems in macrophage membranes [25] can constitute a further obstacle for AZT penetration in these cells. Significantly high concentrations of AZT therefore appear necessary in the bloodstream in order to allow the drug to reach and maintain a minimum effective concentration in the CNS. The intravenous administration of high doses of AZT allowed its detection in the CSF of rats [114], a phenomenon probably due to the saturation of AET systems. Indeed, the AZT amounts detected in the CSF increased when AZT was intravenously co-administered with probenecid [114], even if it is known that the concomitant presence of high hematic AZT and AET inhibitor concentrations cause severe unwanted effects [27,115].

The first studies on nasal administration of zidovudine to rats were performed using formulations constituted by an aqueous suspension of AZT (20 µmol/kg) or a suspension of AZT and probenecid [114]. Following nasal administration, AZT showed rapid absorption in the bloodstream and the CSF, even if the CSF/plasma ratio was lower than 1 and weakly increased in the presence of probenecid [114].

A thermoreversible gel, based on Poloxamer 407 (thermoreversible gelling agent) and n-tridecyl-*β*-d-maltoside (permeation enhancer), was prepared as a nasal formulation of AZT. Doses of 1 mg/kg AZT nasally administered to rabbits led to sensibly higher CSF concentrations of AZT than those obtained by intravenous administration. In particular, the drug targeting efficacy (DTE) and nose-to-brain drug direct transport percentage (DTP) values were 11.51 and 99.27, respectively. Moreover, following the nasal administration of the thermoreversible gel, the CSF/plasma ratio values of AZT were >1 and showed DTE and DTP values of 139.15 and 99.48, respectively [116,117]. These data evidence the importance of appropriate nasal formulations in order to promote the antiviral target in the CNS.

#### 8.3.2. Nasal Formulations for the Administration of a Prodrug of Zidovudine

Recently, the prodrug UDCA-AZT, obtained by means of ester conjugation of zidovudine with ursodeoxycholic acid [71], was loaded in micro-particulate nasal formulations. This prodrug is characterized by its ability to elude the AET systems [71], so it appears to be promising in order to prolong its permanence upon targeting in the central nervous system and to permeate in its macrophages where the AZT activity is required against HIV. We have indeed demonstrated that UDCA-AZT is able to permeate and remain in murine macrophages with an efficiency twenty times higher than that of AZT [25].

Microparticles based on stearic acid were loaded with UDCA-AZT and nasally administered to rats in doses of 800 µg/Kg. The microparticles induced a significant increase of the dissolution rate of the free prodrug, allowing for an efficient uptake in the CSF of rats, but not in the bloodstream, demonstrating the existence of a direct nose—CNS pathway for UDCA-AZT. In the presence of chitosan, the CSF prodrug uptake induced by the stearic acid microparticles increased six times, up to 1.5 µg/mL within 150 min after nasal administration [73]. Taking into account chitosan’s ability to promote UDCA-AZT uptake in the CNS after nasal administration, a new nasal formulation was prepared where the prodrug was encapsulated in chitosan chloride microparticles. In this case too, a selective uptake of UDCA-AZT was obtained, showing concentrations of up to 3 µg/mL in the CSF, where the prodrug can act as an AZT carrier in macrophages [25].

#### 8.3.3. Can Nasal Administration of Insulin be Useful against AIDS Neurotoxicity?

Very recently, it has been demonstrated that insulin treatment of HIV-infected microglia cultures induces a reduction in viral replication as evidenced by the suppression of supernatant HIV-1 p24 levels and by the reduction of CXCL10 and IL-6 transcript levels. Moreover, it has been demonstrated that primary human neurons treated with insulin prevent HIV-1 Vpr-mediated cell process retraction and death. Bearing these aspects in mind, feline immunodeficiency virus (FIV)-infected animals were treated by intranasal insulin and showed reduced CXCL10, IL-6, and FIV RNA levels in brain in comparison with control infected animals. Moreover, nasal administration of insulin allowed for the improvement of neurobehavioral performance in FIV-infected animals, such as motor and memory performances [118]. These data suggest that nasal administration of insulin may represent a new therapeutic option for patients affected by neurodegenerative syndrome HIV-associated neurocognitive disorders.

## 9. Conclusions

We have evidenced that several viruses can easily infect the central nervous system, but even if numerous antiviral therapies can be efficacious at peripheral levels, they appear to be inefficacious at the central level, since the antiviral drug substrates of the active efflux transporters (AET) are expressed by the BBB and macrophages. Exposure of the body to antiviral drugs can further increase the expression of these transporters with a consequent further reduction in the antiviral efficacy in the central nervous system. In order to counteract this phenomenon, the use of AET inhibitors is not allowed in clinical trials, because these inhibitors induce severe unwanted effects when not selectively targeted in specific action sites of drugs. As a consequence, innovative devices and formulations are required in order to selectively target the antiviral drugs and, possibly, the AET inhibitors in the CNS. Several prodrugs of antiviral drugs appear able to inhibit or elude the AET systems, so they would appear to be interesting substrates for innovative formulations. The nasal approach seems to offer a direct nose-to-brain pathway for antiviral drugs. Appropriate devices and formulations, implemented with absorption enhancers, have been designed and administered in order to target the antiviral drugs or their prodrugs in the central nervous system. The results obtained from these studies indicate that the “nasal” strategy is a promising means to promote the efficacy of antiviral therapies against the neurotoxicity of viruses.

## Figures and Tables

**Table 1:** The ATP-binding cassette (ABC) efflux active transporters currently known to interact with antiviral drugs.

Transporter	Name	Gene Symbol	Substrates	Antiviral Substrates	Inhibitors
P-glycoprotein	P-gp	ABCB1	amphipatic cations and organic compounds	saquinavir, ritonavir, lopinavir, amprenavir, nelfinavir, indinavir, abacavir, dolutegravir	cyclosporine-A, verapamil, mefloquine
Multidrug Resistance Protein	MRP-1	ABCC1	hydrophilic anion compounds, large molecules	saquinavir, ritonavir, lopinavir	paclitaxel, probenecid, MK-571
	MRP-4 MRP-5	ABCC4 ABCC5	small polar compounds, nucleoside analogues	Zidovudine, didanosine	
Breast-Cancer-Resistance Protein	BRCP	ABCG2	partially overlap with those of P-gp	zidovudine, lamivudine, abacavir, zalcitabine, stavudine, efavirenz, dolutegravir	ritonavir, saquinavir, nelfinavir
